# Effect of increased positive end-expiratory pressure on intracranial pressure and cerebral oxygenation: impact of respiratory mechanics and hypovolemia

**DOI:** 10.1186/s12868-021-00674-9

**Published:** 2021-11-25

**Authors:** Han Chen, Xiao-Fen Zhou, Da-Wei Zhou, Jian-Xin Zhou, Rong-Guo Yu

**Affiliations:** 1 Fujian Shengli Clinical Medical College, Fujian Medical University, Fuzhou, China; 2 Department of Critical Care Medicine, Fujian Provincial Hospital, Fuzhou, China; 3 Department of Critical Care Medicine, Beijing Tiantan Hospital, Capital Medical University, Beijing, China

**Keywords:** Positive end-expiratory pressure, Intracranial pressure, Cerebral oxygenation, Cerebral blood flow, Respiratory mechanics, Hypovolemia

## Abstract

**Background:**

To evaluate the impact of positive end-expiratory pressure (PEEP) on intracranial pressure (ICP) in animals with different respiratory mechanics, baseline ICP and volume status.

**Methods:**

A total of 50 male adult Bama miniature pigs were involved in four different protocols (n = 20, 12, 12, and 6, respectively). Under the monitoring of ICP, brain tissue oxygen tension and hemodynamical parameters, PEEP was applied in increments of 5 cm H_2_O from 5 to 25 cm H_2_O. Measurements were taken in pigs with normal ICP and normovolemia (*Series I*), or with intracranial hypertension (via inflating intracranial balloon catheter) and normovolemia (*Series II*), or with intracranial hypertension and hypovolemia (via exsanguination) (*Series III*). Pigs randomized to the control group received only hydrochloride instillation while the intervention group received additional chest wall strapping. Common carotid arterial blood flow before and after exsanguination at each PEEP level was measured in pigs with intracranial hypertension and chest wall strapping (*Series IV*).

**Results:**

ICP was elevated by increased PEEP in both normal ICP and intracranial hypertension conditions in animals with normal blood volume, while resulted in decreased ICP with PEEP increments in animals with hypovolemia. Increasing PEEP resulted in a decrease in brain tissue oxygen tension in both normovolemic and hypovolemic conditions. The impacts of PEEP on hemodynamical parameters, ICP and brain tissue oxygen tension became more evident with increased chest wall elastance. Compare to normovolemic condition, common carotid arterial blood flow was further lowered when PEEP was raised in the condition of hypovolemia.

**Conclusions:**

The impacts of PEEP on ICP and cerebral oxygenation are determined by both volume status and respiratory mechanics. Potential conditions that may increase chest wall elastance should also be ruled out to avoid the deleterious effects of PEEP.

**Supplementary Information:**

The online version contains supplementary material available at 10.1186/s12868-021-00674-9.

## Background

Mechanical ventilation is frequently needed in the management of patients with acute intracranial hypertension [1]; however, physicians often confront a “lung and brain dilemma,” where mechanical ventilation, on the one hand, aims at maintaining adequate oxygenation and on the other hand may potentially impair intracranial pressure (ICP) and cerebral blood perfusion. It is still unclear how positive end-expiratory pressure (PEEP) ultimately affects the ICP and cerebral oxygenation. Several studies investigated the impact of elevated PEEP on ICP, which yielded conflicting results. ICP can increase [2–8], not markedly change [9–11], or even decrease [12] after an elevation of PEEP. Several possible determinants for the effect of PEEP on ICP have been proposed, including intracranial compliance, cerebral autoregulation, CO_2_, intracranial Starling resistor, respiratory system elastance (E_RS_) and, in the clinical setting, concomitant medical management [13].

A few studies investigated the potential role of E_RS_ [3, 10, 11, 14]. In theory, increased PEEP would cause an elevation in intrathoracic pressure and diminished venous return from the brain [15–17]. Cerebral blood volume would increase due to the diminished venous return, and ICP would thus increase [18]. Here, the transmission of PEEP into the thoracic cavity is determined by respiratory mechanics: it has been shown that increased lung elastance (E_L_) and decreased chest wall elastance (E_CW_) can minimize the effect of PEEP on pleural pressure [19]. It is worth noting that both E_L_ and E_CW_ determine E_RS_. In other words, an increased E_RS_ may attribute to either the increase in E_L_ due to pulmonary diseases such as acute respiratory distress syndrome (ARDS), or the rise in E_CW_ due to chest wall impairment such as intra-abdominal hypertension, or both [20]. E_L_ and E_CW_ were not differentiated in previous studies [3, 10, 11, 14].

Our group explored the impact of PEEP on ICP under different conditions of elevated E_RS,_ which attribute to high E_L_, E_CW,_ or both [21]. We found that respiratory mechanics determine the effect of PEEP on ICP: in pigs with normal ICP, an increase of PEEP resulted in increased ICP, where the influence was more evident in the condition of increased E_CW_. However, no inter-group (i.e., increased E_CW_ vs*.* normal E_CW_) difference was observed in pigs with elevated baseline ICP. In addition, because of the severe hypotension induced by high PEEP, ICP decreased (rather than increased) in the intracranial hypertension conditions. Due to the nature of a preliminary study, several important factors were not controlled in that study (e.g., non-random design, PaCO_2_ not manipulated, unable to measure cerebral blood flow, etc.). In a series of randomized-controlled experimental studies, we re-evaluate the impact of PEEP on ICP in animals with different respiratory mechanics, baseline ICP and volume status.

## Methods

Comprehensive methods are available (Additional file 1). Male adult Bama miniature pigs (weight 35–40 kg) were used. Animals were purchased from Guangxi University. Humane care was given in compliance with the National Institutes of Health guidelines of experimental animals. By the end of each experiment, the animal was euthanized by overdose pentobarbital (intravenous injection of 120 mg/kg of pentobarbital). The protocol was approved by the Institutional Review Board and Institutional Animal Care and Use Committee of Fujian Provincial Hospital (Approval # KY-2016010).

### The different impacts of increased PEEP on ICP and cerebral oxygenation in animals with different respiratory mechanics where the ICP is normal—*Series I*

Central venous and femoral arterial lines were placed for hemodynamical monitoring and fluid administration. Animals received a tracheotomy and continuous sedation and paralysis. One burr hole was created for ICP and brain tissue oxygen tension (P_ti_O_2_) monitoring. An esophageal catheter was inserted to measure esophageal pressure (i.e., the surrogate of intrathoracic pressure). ARDS (i.e., E_L_ increased) was induced via instillation of 0.1 mol/L hydrochloride down the endotracheal tube. E_CW_ was raised by chest wall strapping [21]. Animals were ventilated (VT 10 mL kg^−1^; FiO_2_ 1.0; PEEP 5 cmH_2_O; the rate was initially set to 20 min^−1^ and adjusted to maintain a PaCO_2_ between 35-45 mmHg) and randomized to chest wall strapping or control group (10 per group). PEEP was gradually increased from 5 to 25 cmH_2_O in a 5 cmH_2_O interval. Hemodynamical parameters, respiratory mechanics, ICP, and P_ti_O_2_ were measured in each PEEP setting.

### The different impacts of increased PEEP on ICP and cerebral oxygenation in animals with different respiratory mechanics where the ICP is elevated—*Series II*

A second burr hole was created, and a Foley catheter was inserted in this series. ICP was increased by inflating the balloon with saline at a rate of 0.5 mL/min. The target was a stable ICP level between 25 and 30 cmH_2_O for > 30 min. Animals were also randomized to chest wall strapping or control group (6 per group), and the same data were collected as in *Series I.*

### The impact of PEEP in the condition of blood volume depletion—Series III

The preparation was as in *Series II*, except that animals were exsanguinated before randomization. The target of exsanguination was a decrease in cardiac output (CO) by ≥ 20%. Animals were then also randomized (6 per group), and the same data were collected as in *Series I and II.*

### The different impacts of PEEP on cerebral perfusion between normal and depleted blood volume—Series IV

In this before-after comparison, hemodynamical monitoring and ventilation settings were as in *Series I*. ARDS was induced, and the chest wall was strapped in all animals (6 animals). ICP monitor and Foley catheters were inserted to produce intracranial hypertension as in *Series II*. Ultrasonography was used to measure common carotid arterial blood flow: an averaged velocity (V) was measured over an entire respiratory cycle. Vessel diameter (D) was measured at the locations where the flow velocity was measured in M-mode. The blood flow passing the measurement location was calculated as 0.25*V*π*D^2^. PEEP was stepwise increased and measurements were taken. Animals were then exsanguinated as in *Series III*. PEEP was increased again, and the blood flow measurements were repeated.

### Analysis

All continuous data were tested for normality of distribution (Shapiro-Wilk) and equal variance, as appropriate. Groups were compared using Mann-Whitney (Rank Sum) tests, and changes over time were examined using Friedman RM-ANOVA on ranks where group distribution was not normal. Where distribution was normal and variance equal, two-way ANOVA or Student’s *t*-test was used as appropriate. Significance was established at *p* < 0.05. Analyses were performed with SPSS Statistics software and GraphPad Prism.

## Results

Details of respiratory mechanics, hemodynamical parameters and blood gas analysis were shown in Additional file 2. E_CW_ was significantly increased by chest wall strapping, while no difference in E_L_ was observed between the control and chest wall strapping groups (Additional file 2: Tables S1–S3).

### Series I

**Fig. 1:**
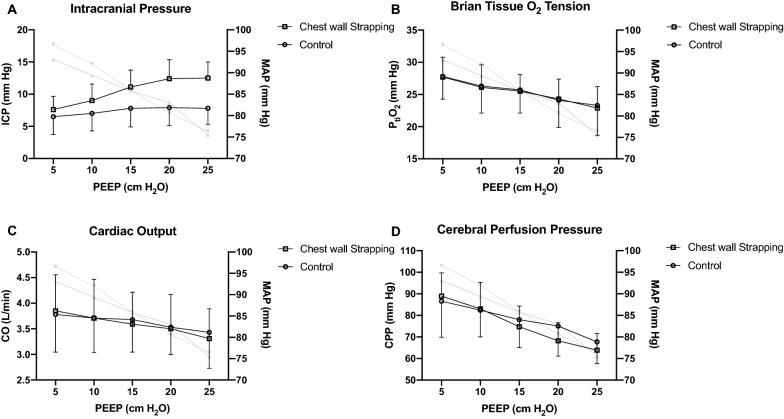
The impacts of positive end-expiratory pressure in animals with normovolemia and normal intracranial pressure (*Series I*, n = 10 per group): Data were presented as mean and standard deviation. RM-ANOVA was used. The corresponding changes of mean arterial pressure were presented in grey lines (showing means only), solid line: control group, dash line: chest wall strapping group. **A** ICP significantly increased when PEEP was increased (*p* < 0.001 for both groups), and the magnitude was significantly higher in the chest wall strapping group than in the control group (*p* = 0.014). **B** P_ti_O_2_ significantly decreased when PEEP was increased (*p* < 0.001 for both groups), but the magnitude was similar between groups (*p* = 0.927). **C** CO significantly decreased with PEEP increment (*p* < 0.001 for both groups), while no difference was observed between groups (*p* = 0.907). **D** CPP significantly decreased with PEEP increment (*p* < 0.001 for both groups), no difference was observed between groups (*p* = 0.645). *ICP* intracranial pressure, *PEEP* positive end-expiratory pressure, *P*_ti_*O*_2_ brain tissue O_2_ tension, *CO* cardiac output, *CPP* cerebral perfusion pressure

ICP increased significantly when PEEP was increased (*p* < 0.001 for both groups), and the magnitude was significantly higher in the chest wall strapping group (*p* = 0.014; Fig. 1A). P_ti_O_2_ decreased significantly when PEEP was increased (*p* < 0.001 for both groups), but the magnitude was similar between groups (*p* = 0.927; Fig. 1B). CO and cerebral perfusion pressure (CPP; calculated as mean arterial pressure minus ICP) decreased with PEEP increment (*p* < 0.001 for both groups), while no difference was observed between groups (*p* = 0.907 and 0.645, respectively; Fig. 1C, D). CO, blood pressure and CPP were preserved in a “physiological” range in each PEEP level.

### Series II

**Fig. 2:**
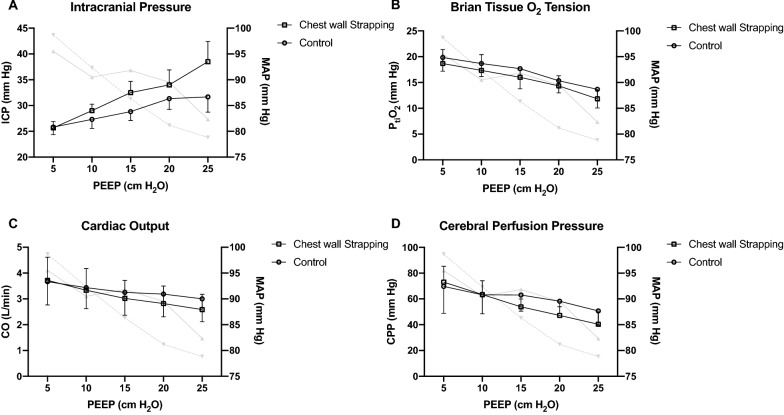
The impacts of positive end-expiratory pressure in animals with normovolemia and intracranial hypertension (*Series II*, n = 6 per group). Data were presented as mean and standard deviation. RM-ANOVA was used. The corresponding changes of mean arterial pressure were presented in grey lines (showing means only), solid line: control group, dash line: chest wall strapping group. **A** ICP significantly increased when PEEP was increased (*p* < 0.001 for both groups), and the magnitude was significantly higher in the chest wall strapping group than in the control group (*p* = 0.022).** B** P_ti_O_2_ significantly decreased when PEEP was increased (*p* < 0.001 for both groups), but the magnitude was similar between groups (*p* = 0.333). **C** CO significantly decreased with PEEP increment (*p* < 0.001 for both groups), while no difference was observed between groups (*p* = 0.649). **D** CPP significantly decreased with PEEP increment (*p* < 0.001 for both groups), no difference was observed between groups (*p* = 0.367). *ICP* intracranial pressure, *PEEP* positive end-expiratory pressure, *P*_ti_*O*_2_ brain tissue O_2_ tension, *CO* cardiac output, *CPP* cerebral perfusion pressure

Similar to *Series I*, the increment of PEEP resulted in a greater elevation of ICP in the chest wall strapping group (*p* = 0.022; Fig. 2A). P_ti_O_2_ decreased significantly when PEEP was increased (*p* < 0.001 for both groups), but no difference was observed between the two groups (*p* = 0.333; Fig. 2B). CO and CPP decreased when PEEP was increased (*p* < 0.001 for both groups), while no inter-group difference was observed (*p* = 0.649 and 0.367, respectively; Fig. 2C, D). CO and blood pressure can be maintained in a “physiological” range, while CPP was lower than 60 mmHg when PEEP was ≥ 20 cmH_2_O (Fig. 2D).

### Series III

**Fig. 3:**
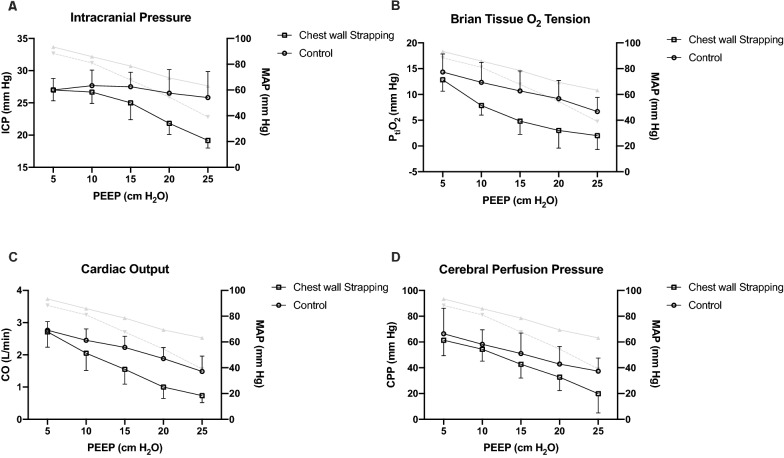
The impacts of positive end-expiratory pressure in animals with hypovolemia and intracranial hypertension (*Series III*, n = 6 per group). Data were presented as mean and standard deviation. RM-ANOVA was used. The corresponding changes of mean arterial pressure were presented in grey lines (showing means only), solid line: control group, dash line: chest wall strapping group. **A** ICP significantly decreased when PEEP was increased (*p* < 0.001 for both groups), and the magnitude was significantly greater in the chest wall strapping group than in the control group (*p* = 0.018). **B** P_ti_O_2_ decreased when PEEP was increased (*p* < 0.001 for both groups), and the magnitude was greater in the chest wall strapping group than in the control group (*p* = 0.020). **C** CO significantly decreased with PEEP increment (*p* < 0.001 for both groups), there was a significantly greater decrease in CO in the chest wall strapping group than in the control group (*p* = 0.020). **D** CPP significantly decreased with PEEP increment (*p* < 0.001 for both groups), no difference was observed between groups (*p* = 0.205). *ICP* intracranial pressure, *PEEP* positive end-expiratory pressure, *P*_ti_*O*_2_ brain tissue O_2_ tension, *CO* cardiac output, *CPP* cerebral perfusion pressure

In the contrast of *Series I* and *II*, the increment of PEEP caused a decrease of ICP when animals were exsanguinated (*p* < 0.001 for both groups). A significantly greater decrease was observed in the chest wall strapping group (*p* = 0.018; Fig. 3A). P_ti_O_2_ decreased when PEEP was increased (*p* < 0.001 for both groups), and the magnitude was greater in the chest wall strapping group (*p* = 0.020; Fig. 3B). CO and CPP also decreased when PEEP was increased (*p* < 0.001 for both groups). There was a significantly greater drop in CO in the chest wall strapping group (*p* = 0.020; Fig. 3C). No significant difference was observed in CPP (*p* = 0.205; Fig. 3D). CPP was lower than 60 mmHg when PEEP was ≥ 15 cmH_2_O.

### Series IV

**Fig. 4:**
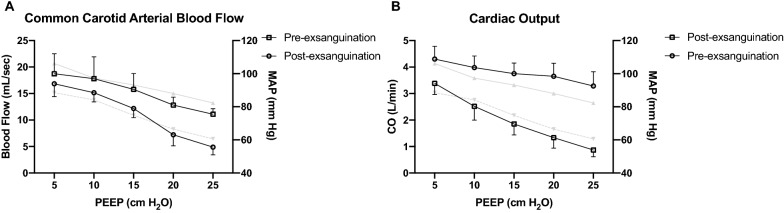
The impacts of positive end-expiratory pressure on common carotid arterial blood flow (*Series IV*, n = 6). Data were presented as mean and standard deviation. RM-ANOVA was used. The corresponding changes of mean arterial pressure were presented in grey lines (showing means only), solid line: pre-exsanguination, dash line: post-exsanguination. **A** Common carotid arterial blood flow decreased significantly when PEEP was increased (*p* < 0.001 for both conditions), and the magnitude was significantly greater in the post-exsanguination (i.e., hypovolemic) condition than in the pre-exsanguination (i.e., normovolemic) condition (*p* < 0.001). **B** CO significantly decreased with PEEP increment (*p* < 0.001 for both conditions), there was a significantly greater decrease in CO in the post-exsanguination condition than in the pre-exsanguination condition (*p* < 0.001). *PEEP* positive end-expiratory pressure, *CO* cardiac output

Compare to baseline, exsanguinated resulted in a greater drop of CO to the same increment of PEEP (*p* < 0.001; Fig. 4B). A significant greater drop of common carotid arterial blood flow was observed following the decrease of CO (*p* < 0.001; Fig. 4A).

## Discussion

The main findings of our study were: (1) ICP was elevated by increased PEEP in both normal ICP and intracranial hypertension conditions in animals with normal blood volume (*Series I* and *II*); (2) Increasing PEEP resulted in decreased ICP if the animals were volume-depleted (*Series III*); (3) Increasing PEEP resulted in decrease in P_ti_O_2_ in either normovolemic or hypovolemic conditions (*Series I*–*III*); (4) The impact of PEEP on hemodynamical parameters, ICP and P_ti_O_2_ became more evident when E_CW_ was increased (*Series I*–*III*); (5) Compare to normovolemic condition, common carotid arterial blood flow was further lowered when PEEP was raised in the condition of hypovolemia, which was probably the cause of lowered ICP and P_ti_O_2_ (*Series IV*).

As shown by Chapin et al., the transmission of airway pressure to pleural pressure depends on the relative compliance of the lung and the chest wall. The transmission is less “efficient” with increased E_L_ and decreased E_CW_ [19]. This means that the applied PEEP in ARDS (a common condition of increased E_L_) is less likely to be transmitted into the pleural space, and thus has less impact on central venous pressure and cerebral venous return. In other words, although a high PEEP is usually applied in the treatment of ARDS, the pulmonary disease per se might be a “protective” factor against the deleterious effects of PEEP to the brain. However, E_L_ is not routinely measured in clinical settings; instead, E_RS_ was often considered synonymous of E_L_. One should keep in mind that E_RS_ and E_L_ are not interchangeable because E_CW_ is also a key determinant of E_RS_. Our data from *Series I* to *III* clearly demonstrated that an increased E_CW_ resulted in a more considerable influence of PEEP on ICP. Our finding suggests that the possible underlining conditions which can increase E_CW_ (e.g., intra-abdominal hypertension due to gastrointestinal retention, or the need for rib fixation due to traumatic rib fractures, which are not rare in clinical settings) must be ruled out before a high PEEP is applied in brain-injured patients.

In contrast to the normovolemic conditions (*Series I* and *II*), raising PEEP resulted in decreased ICP if the animals were volume-depleted (*Series III*). This can be explained by the influence of PEEP on CO and thus cerebral perfusion. As proposed in a conceptual framework that the two important determinants of the impact of elevating PEEP on ICP are the intracranial compliance and the “net” change of cerebral blood volume (CBV); the latter is determined by the balance between the arterial inflow (regulated by CO, cerebral autoregulation, PaCO_2_, etc.) and venous outflow (regulated by PEEP, Starling resistor, etc.). Increased PEEP and subsequent increased pleural pressure impede the global venous return to the heart, lead to a decrease in CO and cerebral perfusion [22, 23]. The effect is more profound in the volume-depleted condition [24], as was observed in *Series III*. In this case, the influence of decreased CO (which reduces arterial inflow and decreases CBV) exceeded the influence of decreased venous return (which reduces venous outflow and increases CBV), which resulted in a negative “net” change of CBV and eventually a decreased ICP.

Mean arterial pressure (MAP) and CPP were also lower when high PEEP levels were applied. In animals with intact cerebral autoregulation, vessels in the brain can maintain a constant cerebral blood flow via regulating the vascular tone throughout a wide range of MAP or CPP [25, 26]. In the present study, CPP dropped beyond the lower limit of autoregulation when high PEEP levels were selected in animals received exsanguination (*Series III*). Therefore, the most possible cause of lowered ICP and P_ti_O_2_ was decreased cerebral perfusion. More data was obtained to support the concept in *Series IV*. We measured the blood flow in the common carotid artery and a significantly greater decrease of blood flow was observed after exsanguination when the same PEEP was applied. Take all data together, we can conclude that PEEP can affect ICP predominantly via its hemodynamical effects, which always functions on both the arterial (perfusion) and venous (returning) sides simultaneously. And here, increased E_CW_ amplifies the effects of PEEP.

P_ti_O_2_ was also measured in the present study, and we found that increasing PEEP resulted in decreased P_ti_O_2_ in either normovolemic or hypovolemic conditions. The impact of PEEP on cerebral oxygenation was not well understood yet. Results from previous studies are variable [3, 9, 27]. In traumatic brain injury patients with ARDS, a progressively raising of PEEP (from 5 to 15 cmH_2_O) improved P_ti_O_2_ [9]. In an experimental study, a raising-PEEP strategy was carried out in healthy pigs where no impact on P_ti_O_2_ was observed. The conflicts of our data to previous studies can be explained as follows: assuming a relatively constant O_2_ consumption, the change of P_ti_O_2_ should be the consequence of changed O_2_ delivery. Thus, the two key determinants of O_2_ delivery to the brain were CO and arterial O_2_ saturation. Unlike Nemer’s study where increasing PEEP resulted in a considerable improvement in P/F ratio (from 108.5 to 203.6 mmHg), the improvement in P/F ratio was less obvious (although statistically significant, Additional file 1: Fig. S1–S3) in the present study; moreover, O_2_ saturation was 100% already and thus further increase was not possible since the FiO_2_ was 1.0 in the first place. On the other hand, CO was decreased following PEEP increments. Therefore, P_ti_O_2_ decreased due to a decreased CO.

It is also interesting to know the influence of increased PEEP on ICP and other parameters in animals with normal ICP (like in *Series I*) but with depleted blood volume (like in *Series III*). In fact, we did perform some pilot experiments to exam this. Briefly, a crossover rather than a randomized-control experiment was completed in four pilot animals with normal ICP and depleted blood volume (two from chest wall strapping to control condition and the other two in the inverse order). ICP decreased with the increment of PEEP in all animals, but the magnitude was small (from 8.5 to 6.8 mmHg in the control condition and from 7.1 to 4.8 mmHg in the chest wall strapping condition with PEEP increment, Additional file 2: Fig. S4). The reason we did not further perform a randomized-control study with a larger sample size were: first, the trend of ICP change in the pilot study was the same as we observed in *Series III*, and the underlying pathophysiology rationale could be the same; second, considering the small difference in the change of ICP between the two conditions, large sample size is required to obtain a statistical significance, which may deviate from the ethical requirements of animal experiments. Therefore, we finished only one randomized-control study (*Series III*) to exam the impact of hypovolemia and speculated that the conclusion might also be true in animals with normal ICP, although without additional experiments.

Our study has some limitations: (1) The data were obtained with experiments of short duration; therefore, the long term effects of PEEP were still unclear; (2) Blood flow was measured at the common carotid artery rather than the internal carotid artery (which perfuse the brain). In the present study, we used a Vevo 3100 ultrasonic system with an ultra-high-frequency probe designed for small animal research. The ultra-high-frequency probe provided high resolution while the maximal scan depth was limited. The internal carotid artery was too deep to display by using the current device, so we chose common carotid artery instead; (3) Although a decreased cerebral venous return was proposed as the main reason for increased ICP, the outflow was not measured in this study. We measured arterial blood flow via Doppler ultrasound; however, using the same methodology to assess cerebral venous return was suggested to be inaccurate [28]; (4) Indeed, ICP has four components: besides arterial blood inflow and venous blood outflow discussed above, cerebrospinal fluid (CSF) circulation and volumetric changes of brain tissue or contusion volume also play an important role. We did not directly measure the change of CSF circulation and brain tissue volume in the present study. Considering the short experimental period, brain tissue volume can be assumed unchanged, while the change of CSF circulation was not investigated in a quantitative way but a qualitative way instead (*Series I* vs.* Series II*). In other words, the four components of ICP were not investigated in isolation and the quantitative way in the present study and thus may limit our interpretation of the effects of a specific factor. Moreover, several other factors that may have an impact on these four components, such as pulse amplitude, respiratory wave amplitude and pressure reactive index (as a surrogate of cerebral autoregulation) were not considered in the present study. (5) The present study investigates only two different volume statuses (normovolemia vs. massive blood loss) while intermediate states, for example, mild hypovolemia due to diuretics or hyperosmolar agents, or small amount of blood loss, are more likely to occur in the clinical setting. Further studies are necessary to confirm the effects of PEEP in these situations.

## Conclusions

Both volume status and respiratory mechanics determine the impacts of PEEP on ICP and cerebral oxygenation. PEEP increases ICP in stable hemodynamic conditions while decreases ICP in hypovolemia. The effects are more profound when E_CW_ is high. In brain-injured patients, PEEP should be applied under the monitoring of MAP, ICP, and CPP; meanwhile, potential conditions that may increase E_CW_ should be ruled out to avoid the deleterious effects of PEEP.

## Supplementary Information

**Additional file 1.** Comprehensive experimental methods.

**Additional file 2.** Details of respiratory mechanics, hemodynamical parameters and blood gas analysis.

## Data Availability

The datasets used and/or analyzed during the present study are available from the corresponding author on reasonable request.

## Abbreviations

ARDS: Acute respiratory distress syndrome
CO: Cardiac output
CPP: Cerebral perfusion pressure
E_CW_: Chest wall elastance
E_L_: Lung elastance
E_RS_: Respiratory system elastance
ICP: Intracranial pressure
MAP: Mean arterial pressure
PEEP: Positive end-expiratory pressure
P_ti_O_2_: Brain tissue oxygen tension
VT: Tidal volume
