# Supplementary material for: Effect of increased positive end-expiratory pressure on intracranial pressure and cerebral oxygenation: impact of respiratory mechanics and hypovolemia
Source: BMC Neurosci. 2021 Nov 25;22:72. doi: 10.1186/s12868-021-00674-9 (PMC8614026; doi:10.1186/s12868-021-00674-9)
Supplement: Supplementary file 1 — Additional file 1. Comprehensive experimental methods. [file 12868_2021_674_MOESM1_ESM.pdf]

**ADDITIONAL FILE 01**

# EFFECT OF INCREASED POSITIVE END-EXPIRATORY PRESSURE ON INTRACRANIAL PRESSURE AND CEREBRAL OXYGENATION – *IMPACT OF RESPIRATORY MECHANICS AND HYPOVOLEMIA*

Han Chen, MD, phD[1,2]; Xiao-Fen Zhou, MD[1,2]; Da-Wei Zhou, MD[3]; Jian-Xin Zhou, MD, phD[3], Rong-Guo Yu, MD, phD[1,2,*]

**Affiliations**: [1]Fujian Provincial Clinical college, Fujian Medical University; [2]Surgical Intensive Care Unit, Fujian Provincial Hospital; [3]Department of Critical Care Medicine, Beijing Tiantan Hospital, Capital Medical University

**Correspondence**: Dr. Rong-Guo Yu (garyyrg@126.com; garyyrg@yahoo.com)

# METHODS

## Animal preparation

Male adult Bama miniature pigs (weight 35-40 kg) were used. Animals were purchased from Guangxi University. All animals received humane care in compliance with the National Institutes of Health guidelines for the care and use of experimental animals. The protocol was approved by the Institutional Review Board of Fujian Provincial Hospital (Approval # KY-2016010).

Animals have fasted preoperatively. Animals were connected to a cardiorespiratory monitor (N19, Mindary Inc., Shenzhen, China), and a rectal probe was placed for continuous temperature monitoring. Premedication consisted of intramuscular 10-20 mg/kg ketamine, followed by an intravenous bolus of 10 mg midazolam. Intravenous infusion of 0.2-1.0 $mg \cdot kg^{-1} \cdot hr^{-1}$ midazolam and 0.1-0.2 $mcg \cdot kg^{-1} \cdot hr^{-1}$ fentanyl were used for sedation. Lactate Ringer's solution was continuously given at the rate of 5 $mL \cdot kg^{-1} \cdot hr^{-1}$; no additional fluids and catecholamine infusions were allowed. A central venous catheter was placed via the right internal jugular vein for fluid infusion and central venous pressure (CVP) measurements. Arterial cannula was placed via the right femoral artery to measure blood pressure. A Pulse Contour Cardiac Output (PiCCO$^{TM}$) monitoring was used to measure hemodynamical parameters. Cardiac output (CO) was measured by using transpulmonary thermodilution technology: 15 mL of cold saline was rapidly injected via the central venous catheter, and the reading on the monitor was recorded. Three measurements were taken, and the average was calculated.

A tracheotomy was performed, a 6.5# tracheotomy cuffed tube was placed. Animals were then paralyzed (10 mg vecuronium bromide bolus) and mechanically ventilated with a Servo-s ventilator (Maquet, Solna, Sweden) with the following settings: PC 10 $cmH_2O$; $FiO_2$ 1.0; PEEP 5 $cmH_2O$; rate 20 $min^{-1}$. Vecuronium bromide was continuously infused (1 $mg{\cdot}kg^{-1}{\cdot}hr^{-1}$), and an additional 5 mg bolus was administrated if spontaneous breathing effort presented. A SmartCath-G esophageal balloon catheter (7003300, CareFusion Co., Yorba Linda, CA, USA) was placed to measure esophageal pressure ($P_{ES}$). Positive pressure occlusion test was performed – chest wall was compressed and the changes of airway pressure ($P_{AW}$) and $P_{ES}$ were recorded. A $\triangle P_{ES}/\triangle P_{AW}$ ratio between 0.8-1.2 indicated proper balloon position (1, 2). $P_{ES}$ and $P_{AW}$ were measured by two pressure transducers connected to the monitor.

**Experimental Series I – The different impact of increased PEEP on ICP and cerebral oxygenation in animals with different respiratory mechanics where the ICP is normal:** A midline, transverse incision was performed to expose the skull. One burr hole was created approximately 10 mm left/lateral of midline and 10 mm anterior to the coronal suture. An intraparenchymal ICP monitor catheter (Codman Microsensor, Raynham, MA, USA) and a region brain tissue oxygen tension ($P_{ti}O_2$) monitor catheter were placed via this opening. ICP monitor (Codman ICP Express, Raynham, MA, USA) and $P_{ti}O_2$ monitor (Licox®, Integra LifeSciences Ltd., Ireland) were connected. To induce acute respiratory distress syndrome (ARDS, i.e., $E_L$ increased), 0.1 mol/L hydrochloride solution (4 mL/kg) was instilled via a thin suction catheter placed at the level of the carina. ARDS was validated by achieving a

$PaO_2/FiO_2$ ratio of ≤ 100. Animals were then ventilated (VT 10 mL·kg$^{-1}$; $FiO_2$ 1.0; PEEP 5 cm$H_2O$) and respiratory rate was (initially set to 20 min$^{-1}$ and) adjusted to maintain a $PaCO_2$ between 35-45 mmHg. Animals were randomized to chest wall strapping or control group (10 per group). In the chest wall strapping group, an inelastic, adjustable bellyband was strapped around the animals' chest wall and abdomen to increase chest wall elastance. Two pneumatic cuffs were placed between the bellyband and the animal; to increase $E_{CW}$, the cuffs were inflated to a pressure of 20 cm $H_2O$ (3-5). PEEP was gradually increased from 5 to 25 cm$H_2O$ in a 5 cm$H_2O$ interval. Hemodynamical parameters, respiratory mechanics, ICP, $P_{ti}O_2$ and arterial blood gases (ABG) were measured in each PEEP setting. To measure respiratory mechanics, end-inspiratory and end-expiratory occlusion were performed, each for 3 s. $P_{ES}$ and $P_{AW}$ during the last second of occlusion were recorded. Respiratory mechanics were calculated as follows:

$$E_{RS}=\frac{P_{PLAT} - PEEP_{TOTAL}}{V_T}$$

Where $P_{PLAT}$ and $PEEP_{TOTAL}$ represent $P_{AW}$ at end-inspiratory and end-expiratory occlusion, respectively.

$$E_{CW}=\frac{P_{ES\text{-}EI} - P_{ES\text{-}EE}}{V_T}$$

Where $P_{ES\text{-}EI}$ and $P_{ES\text{-}EE}$ are respective $P_{ES}$ determined at end-inspiratory and end-expiratory occlusion.

$E_L = E_{RS} - E_{CW}$

**Experimental Series II – The different impact of increased PEEP on ICP and cerebral oxygenation in animal with different respiratory mechanics where the ICP is elevated:** Animals were prepared as in

***series I.*** One additional burr hole was created approximately 10 mm right/lateral of midline and 10 mm anterior to the coronal suture. A balloon-tipped catheter (5 mL, 8Fr Foley) was placed through the hole for inducing intracranial hypertension in this series. ICP was Increased by inflating the balloon with saline at a rate of 0.5 mL/min until the ICP was constant between 25 and 30 $cmH_2O$ for > 30 min. After stabilization, animals were also randomized to chest wall strapping or control group (6 per group) and the same data were collected as in ***series I***.

**Experimental Series III – The impact of PEEP in the condition of blood volume depletion:** The preparation was as in ***series II***, except that animals were exsanguinated before randomization. The arterial catheter was connected to a clean collecting bag to allow blood loss. The target of exsanguination was a decrease in cardiac output of ≥ 20% of the baseline. Animals were then randomized to either chest wall strapping or control group (6 per group) and the same data were collected as in ***Series I* and *II***.

**Experimental Series IV – The different impact of PEEP on cerebral perfusion between normal and depleted blood volume:** In this before-after comparison, hemodynamical monitoring and ventilation settings were as in ***Series I***. ARDS was induced and chest wall was strapped in all four animals (6 animals). Ultrasonographic imaging was performed using a commercially available system (Vevo 3100, FUJIFILM VisualSonics Inc., USA) to measure common carotid arterial blood flow. Recording of Pulse-Wave Doppler was obtained from the common carotid artery 3 to 4 mm before the bifurcation of the

external and internal carotid artery to measure blood flow velocity. We did not measure internal carotid arterial blood flow because it was not always possible to display internal carotid arterial – a deep and relatively thin structure – in pigs. Doppler velocity measurement was made at the smallest possible angle of incidence between the Doppler beam and the blood flow direction. Because respirations can cause variation of velocity, an averaged velocity (V) was measured over an entire respiratory cycle (i.e., measured from a peak waveform to the next peak one). Vessel diameter (D) was measured at the locations where the flow velocity was measured in M-mode. Three measurements were taken in different cardiac cycles and averaged to overall average vessel diameter. The blood flow passing the measurement location was calculated as $0.25*V*\pi*D^2$. PEEP was stepwise increased and measurements were taken at each PEEP level. Animals were then exsanguinated as in ***Series III***. PEEP was increased again and the blood flow measurements were repeated. By the end of each experiment the animal was euthanized by overdose pentobabital.

**Analysis:** All continuous data were tested for normality of distribution (Shapiro-Wilk) and equal variance, as appropriate. Groups were compared using Mann-Whitney (Rank Sum) tests, and changes over time were examined by using Friedman RM-ANOVA on ranks where group distribution was not normal. Where distribution was normal and variance equal, two-way ANOVA or Student's *t*-test was used as appropriate.  Significance was established at $p < 0.05$. Analyses were performed with SPSS statistics software (V.23.0, IBM Corporation, NY, USA) and GraphPad Prism (V.8.02, GraphPad Software Inc., CA, USA).

**REFERENCES**

1. Chiumello D, Consonni D, Coppola S, et al: The occlusion tests and end-expiratory esophageal pressure: measurements and comparison in controlled and assisted ventilation. Ann Intensive Care 2016; 6:13.

2. Chen H, Yang YL, Xu M, et al: Use of the injection test to indicate the oesophageal balloon position in patients without spontaneous breathing: a clinical feasibility study. J Int Med Res 2017; 45:320-331.

3. Hussain SN, Pardy RL: Inspiratory muscle function with restrictive chest wall loading during exercise in normal humans. J Appl Physiol (1985) 1985; 58:2027-2032.

4. Staffieri F, Stripoli T, De Monte V, et al: Physiological effects of an open lung ventilatory strategy titrated on elastance-derived end-inspiratory transpulmonary pressure: study in a pig model. Crit Care Med 2012; 40:2124-2131.

5. Chen H, Zhou J, Lin YQ, et al: Intracranial pressure responsiveness to positive end-expiratory pressure in different respiratory mechanics: a preliminary experimental study in pigs. BMC Neurol 2018; 18:183.
