# Supplementary material for: Effect of increased positive end-expiratory pressure on intracranial pressure and cerebral oxygenation: impact of respiratory mechanics and hypovolemia
Source: BMC Neurosci. 2021 Nov 25;22:72. doi: 10.1186/s12868-021-00674-9 (PMC8614026; doi:10.1186/s12868-021-00674-9)
Supplement: Supplementary file 2 — Additional file 2. Details of respiratory mechanics, hemodynamical parameters and blood gas analysis. [file 12868_2021_674_MOESM2_ESM.pdf]

ADDITIONAL FILE 02

EFFECT OF INCREASED POSITIVE END-EXPIRATORY PRESSURE ON INTRACRANIAL PRESSURE AND CEREBRAL OXYGENATION – *IMPACT OF RESPIRATORY MECHANICS AND HYPOVOLEMIA*

Han Chen, MD, phD[1,2]; Xiao-Fen Zhou, MD[1,2]; Da-Wei Zhou, MD[3]; Jian-Xin Zhou, MD, phD[3], Rong-Guo Yu, MD, phD[1,2,*]

Affiliations: [1]Fujian Provincial Clinical college, Fujian Medical University; [2]Surgical Intensive Care Unit, Fujian Provincial Hospital; [3]Department of Critical Care Medicine, Beijing Tiantan Hospital, Capital Medical University

Correspondence: Dr. Rong-Guo Yu (garyyrg@126.com; garyyrg@yahoo.com)

Figure E1 - The impacts of positive end-expiratory pressure on hemodynamical and blood gas parameters in animals with normovolemia and normal intracranial pressure (*Series I*)

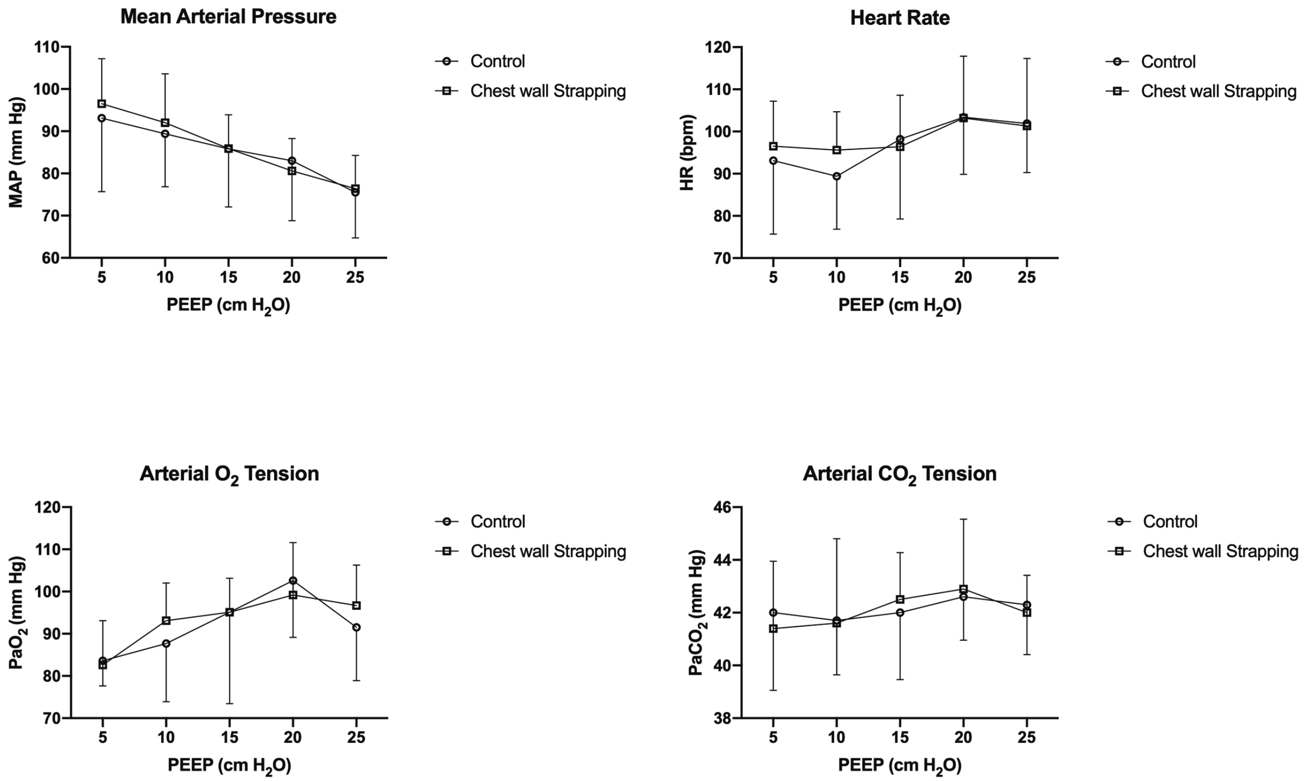

Panel A MAP significantly decreased when PEEP was increased (p < 0.001), but the magnitude was similar between groups (p = 0.848). **Panel B** Heart rate significantly increased when PEEP was increased (p= 0.003), but the magnitude was similar between groups (p = 0.789). **Panel C** PaO_2 significantly (but slightly) increased with PEEP increment (p = 0.002), while no difference was observed between groups (p = 0.729). **Panel D** $PaCO_2$ was manipulated within a normal range at each PEEP level (p = 0.495); no difference was observed between groups (p = 0.941). **Abbreviations** MAP mean arterial pressure, PEEP positive end-expiratory pressure, HR heart rate, PaO_2 arterial O_2 tension, $PaCO_2$ arterial CO_2 tension.

Figure E2 - The impacts of positive end-expiratory pressure on hemodynamical and blood gas parameters in animals with normovolemia and intracranial hypertension (*Series II*)

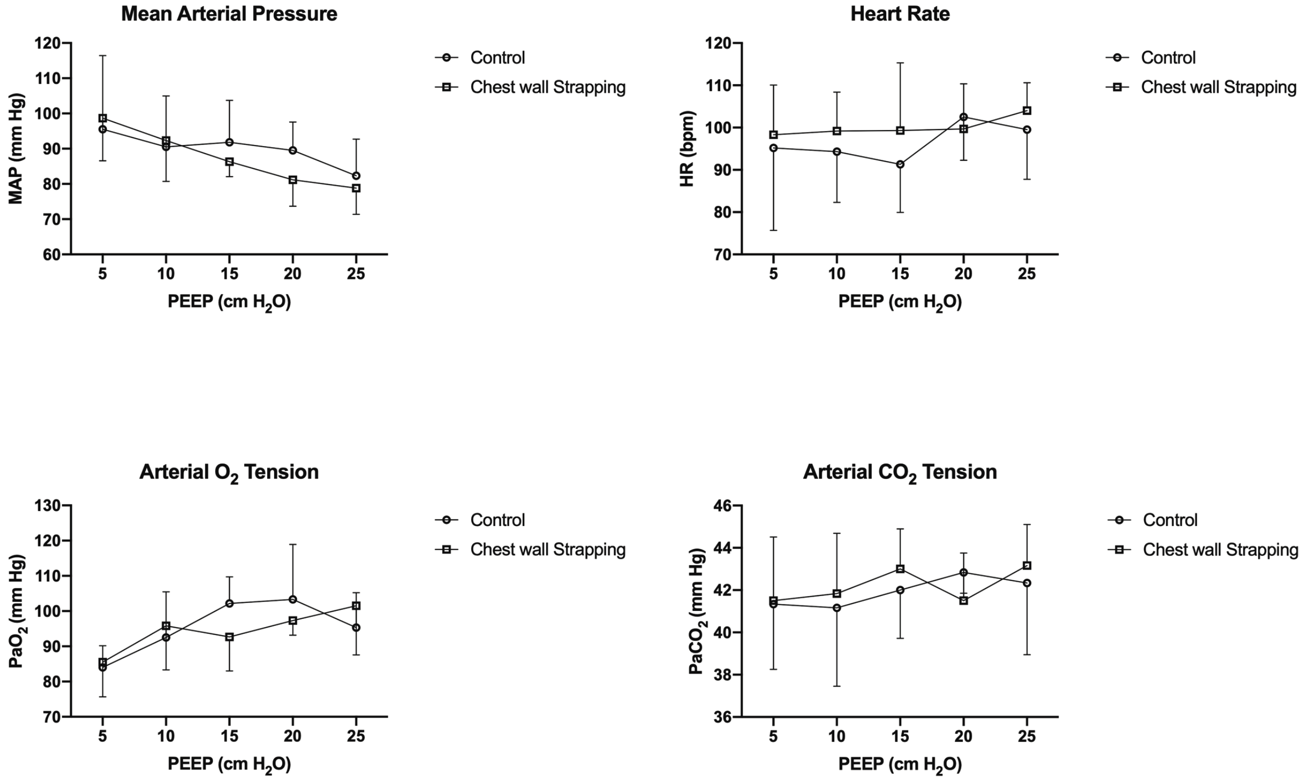

Panel A MAP significantly decreased when PEEP was increased (p = 0.003), but the magnitude was similar between groups (p = 0.667). **Panel B** No difference in heart rate was observed when PEEP was increased (p= 0.188), no difference was observed between groups either (p = 0.570). **Panel C** PaO_2 significantly (but slightly) increased with PEEP increment (p = 0.007), while no difference was observed between groups (p = 0.807). **Panel D** $PaCO_2$ was manipulated within a normal range at each PEEP level (p = 0.480); no difference was observed between groups (p = 0.795). **Abbreviations** MAP mean arterial pressure, PEEP positive end-expiratory pressure, HR heart rate, PaO_2 arterial O_2 tension, $PaCO_2$ arterial CO_2 tension.

Figure E3 - The impacts of positive end-expiratory pressure on hemodynamical and blood gas parameters in animals with hypovolemia and intracranial hypertension (*Series III*)

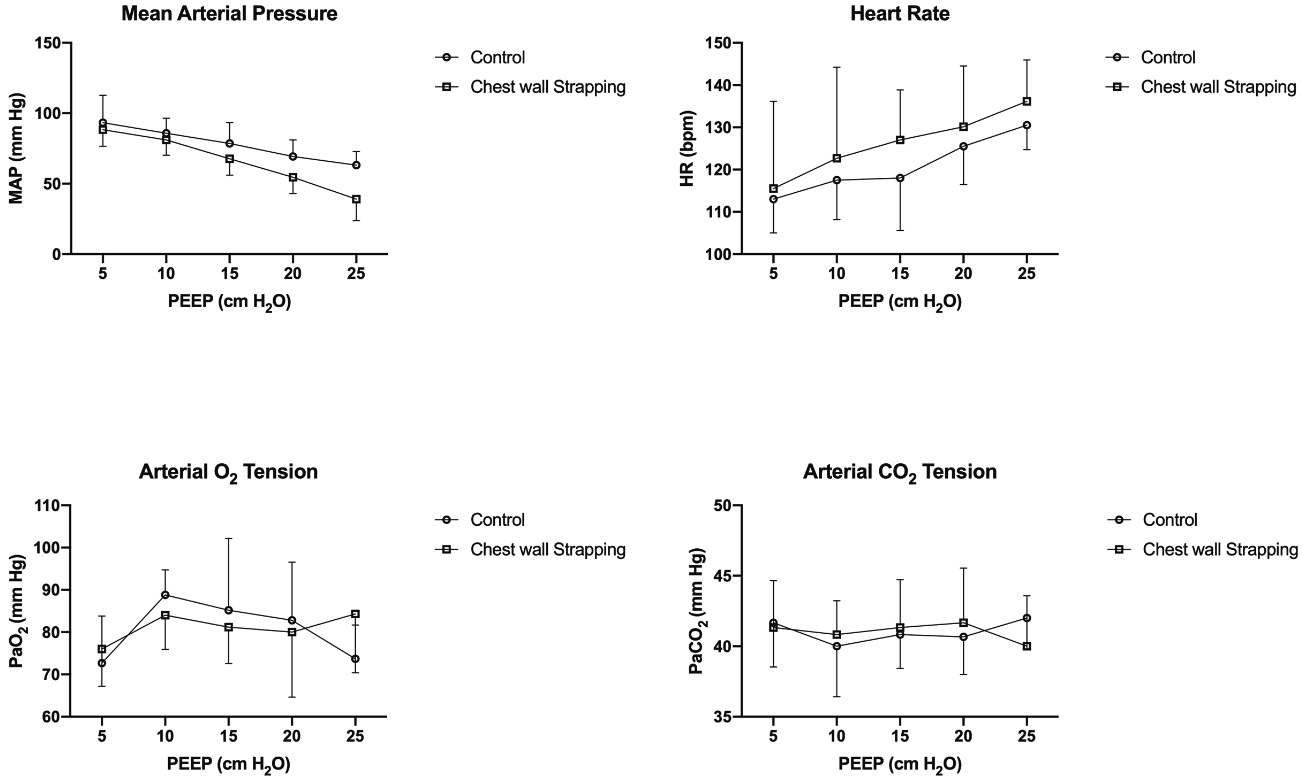

Panel A MAP significantly decreased when PEEP was increased ($p < 0.001$), animals in the chest wall strapping groups has a trend of greater decrease in MAP ($p = 0.096$). **Panel B** Heart rate significantly increased when PEEP was increased ($p = 0.003$), but the magnitude was similar between groups ($p = 0.384$). **Panel C** No difference was observed in PaO_2 with PEEP increment ($p = 0.068$), no difference was observed between groups either ($p = 0.919$). **Panel D** $PaCO_2$ was manipulated within a normal range at each PEEP level ($p = 0.930$); no difference was observed between groups ($p > 0.999$).

Abbreviations MAP mean arterial pressure, PEEP positive end-expiratory pressure, HR heart rate, PaO_2 arterial O_2 tension, $PaCO_2$ arterial CO_2 tension.

Table E1 Respiratory mechanics measured at a positive end-expiratory pressure of 5 cmH_2O in

Series I

	Control (n = 10)	Chestwall Strapping (n = 10)	*p* value
Body weight (kg)	38.7 ± 1.2	38.5 ± 0.6	0.560
Tidal volume (mL)	388 ± 10	386 ± 7	0.618
Respiratory system compliance (mL/cm H_2O)	14.8 ± 2.8	11 ± 1.6	0.002
Peak airway pressure (cm H_2O)	37.2 ± 6.1	44.6 ± 6.2	0.015
Plateau pressure (cm H_2O)	32.4 ± 5.5	40.9 ± 5.5	0.003
Positive end-expiratory pressure (cm H_2O)	5 (5, 6)	5 (5, 5.3)	0.615
End-expiratory esophageal pressure (cm H_2O)	5.7 ± 1.8	8.4 ± 2.1	0.006
End-inspiratory esophageal pressure (cm H_2O)	10.2 ± 2.6	21.6 ± 4.4	<0.001
Respiratory system driving pressure (cm H_2O)	27.1 ± 5.5	35.7 ± 5.4	0.002
Respiratory system elastance (cm H_2O/L)	70 ± 14	92.8 ± 13.6	0.002
Transpulmonary driving pressure (cm H_2O)	22.6 ± 4.8	22.5 ± 4.1	0.945
Lung elastance (cm H_2O/L)	58.4 ± 12.3	58.4 ± 10.5	0.998
Chest-wall driving pressure (cm H_2O)	4.5 ± 1.2	13.2 ± 3	<0.001
Chest-wall elastance (cm H_2O/L)	11.6 ± 3	34.4 ± 7.7	<0.001
E_{CW}/E_{RS} ratio	16.5 (13.0, 19.7)	35.1 (32.9, 38.1)	<0.001

Data presented as mean ± standard deviation or median (interquatile range).

E_{CW}: chest wall elastance; E_{RS}: respiratory system elastance.

Table E2 Respiratory mechanics measured at a positive end-expiratory pressure of 5 cmH_2O in *Series II*

	Control (n = 6)	Chestwall Strapping (n = 6)	*p* value
Body weight (kg)	38.9 ± 1.1	38.6 ± 1.1	0.673
Tidal volume (mL)	390 ± 11	387 ± 12	0.628
Respiratory system compliance (mL/cm H_2O)	15.6 ± 1.3	10.5 ± 0.8	<0.001
Peak airway pressure (cm H_2O)	33.8 ± 2.4	46.5 ± 2.4	<0.001
Plateau pressure (cm H_2O)	30.2 ± 2.5	42 ± 3	<0.001
Positive end-expiratory pressure (cm H_2O)	5 ± 0	5 ± 0	>0.999
End-expiratory esophageal pressure (cm H_2O)	5.6 ± 1.5	9.7 ± 1.9	0.002
End-inspiratory esophageal pressure (cm H_2O)	9.1 ± 2.2	24.5 ± 4.7	<0.001
Respiratory system driving pressure (cm H_2O)	25.2 ± 2.5	37 ± 3	<0.001
Respiratory system elastance (cm H_2O/L)	64.4 ± 5.3	95.9 ± 6.9	<0.001
Transpulmonary driving pressure (cm H_2O)	21.7 ± 2.1	22.3 ± 2.6	0.708
Lung elastance (cm H_2O/L)	55.6 ± 4.4	57.7 ± 7.2	0.548
Chest-wall driving pressure (cm H_2O)	3.5 (2.7, 4.3)	13.8 (11.9, 17.1)	0.004
Chest-wall elastance (cm H_2O/L)	8.8 ± 2.1	38.1 ± 8.8	<0.001
E_{CW}/E_{RS} ratio	13.7 ± 2.7	39.6 ± 7.4	<0.001

Data presented as mean ± standard deviation or median (interquatile range).

E_{CW}: chest wall elastance; E_{RS}: respiratory system elastance.

Table E3 Respiratory mechanics measured at a positive end-expiratory pressure of 5 cmH_2O in *Series III*

	Control (n = 6)	Chestwall Strapping (n = 6)	*p* value
Body weight (kg)	39.2 ± 0.6	38.5 ± 1.1	0.188
Tidal volume (mL)	393.3 ± 5.2	386.7 ± 12.1	0.256
Respiratory system compliance (mL/cm H_2O)	13.4 ± 0.9	10 ± 0.9	<0.001
Peak airway pressure (cm H_2O)	39 ± 3.7	48.7 ± 3.4	0.001
Plateau pressure (cm H_2O)	34.5 ± 2.2	44 ± 3.6	<0.001
Positive end-expiratory pressure (cm H_2O)	5.1 ± 0.2	5 ± 0.1	0.229
End-expiratory esophageal pressure (cm H_2O)	5.7 ± 1.9	9.1 ± 2.6	0.028
End-inspiratory esophageal pressure (cm H_2O)	9.7 ± 1.6	22.9 ± 5.2	<0.001
Respiratory system driving pressure (cm H_2O)	29.4 ± 2.1	39 ± 3.7	<0.001
Respiratory system elastance (cm H_2O/L)	74.7 ± 4.9	100.9 ± 9	<0.001
Transpulmonary driving pressure (cm H_2O)	25.3 ± 1.9	25.3 ± 1.9	0.929
Lung elastance (cm H_2O/L)	64.4 ± 4.5	65.3 ± 4.8	0.751
Chest-wall driving pressure (cm H_2O)	3.9 (3.4, 4.6)	12.8 (11.7, 15.4)	0.004
Chest-wall elastance (cm H_2O/L)	10.3 ± 1.9	35.6 ± 8.5	<0.001
E_{CW}/E_{RS} ratio	13.7 ± 2.3	35 ± 5.5	<0.001

Data presented as mean ± standard deviation or median (interquatile range).

E_{CW}: chest wall elastance; E_{RS}: respiratory system elastance.

Figure E4 Change of ICP with PEEP increment in the crossover pilot experiments.

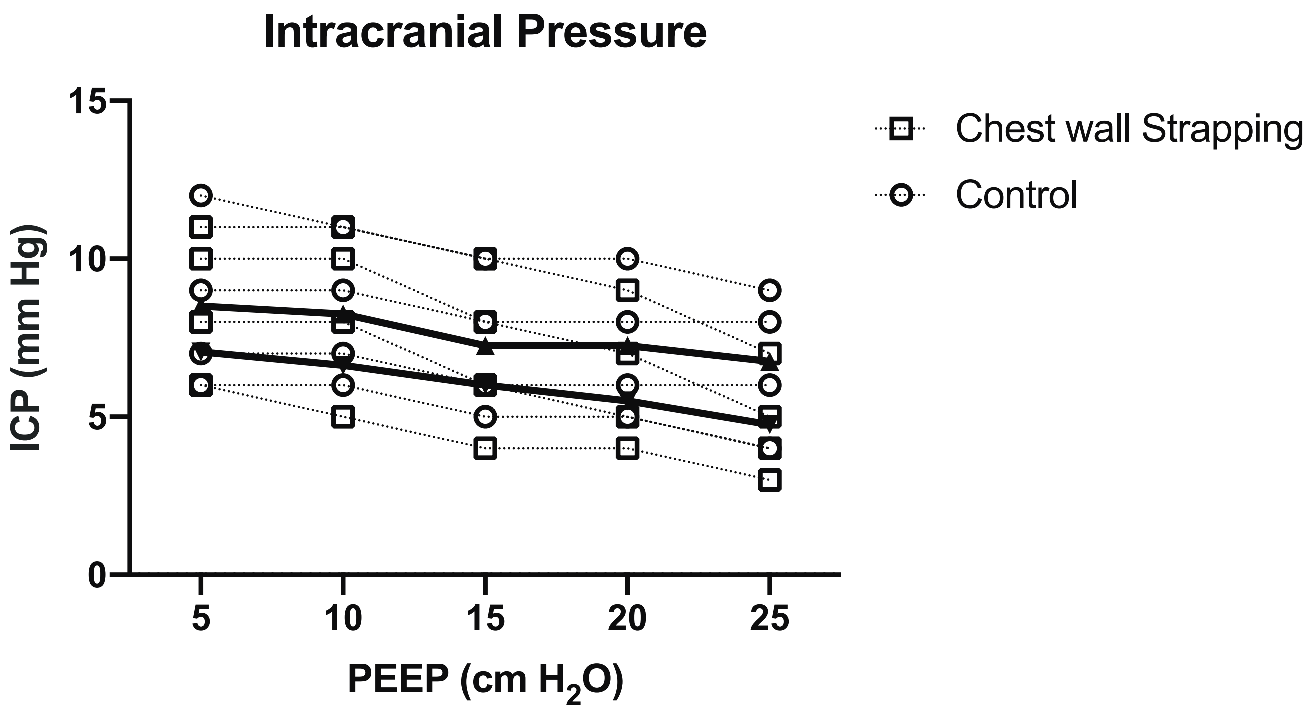

Crossover pilot experiments (n = 4) were conducted to show the impact of increased PEEP on ICP in animals with normal ICP but with depleted blood volume. Individual changes of ICP with PEEP increment were shown. The bold-solid lines represent the mean ICP of chest wall strapping condition (lower, with ▼ symbol) or control condition (upper, with ▲ symbol), respectively. Note that no statistical test was performed. **Abbreviations** ICP intracranial pressure, PEEP positive end-expiratory pressure
